# Low-to-Moderate-Intensity Resistance Exercise Effectively Improves Arterial Stiffness in Adults: Evidence From Systematic Review, Meta-Analysis, and Meta-Regression Analysis

**DOI:** 10.3389/fcvm.2021.738489

**Published:** 2021-10-11

**Authors:** Yong Zhang, Ya-Jun Zhang, Weibing Ye, Mallikarjuna Korivi

**Affiliations:** ^1^Department of Rehabilitation Medicine, College of Medicine, Shaoxing University, Shaoxing, China; ^2^Exercise and Metabolism Research Center, College of Physical Education and Health Sciences, Zhejiang Normal University, Jinhua, China

**Keywords:** pulse wave velocities, arterial stiffness, resistance training, cardiovascular, meta-analyses

## Abstract

**Background/Purpose:** Resistance exercise (RE) is known to improve cardiovascular health, but the role of RE variables on arterial stiffness is inconclusive. In this systematic review and meta-analysis, we investigated the influence of RE and its intensities on arterial stiffness measured as pulse wave velocity (PWV) in young and middle-aged adults.

**Methods:** Web of Science, PubMed/MEDLINE, Scopus, EMBASE, Cochrane Library, ScienceDirect, CINAHL, Wiley Online Library, and Google Scholar were searched for relevant studies. RE trials that reported PWV data, and compared with respective controls were included. The Cochrane Collaboration tool was used to assess the risk of bias.

**Results:** Data were synthesized from a total of 20 studies, involving 981 participants from control (*n* = 462) and exercise (*n* = 519) trials. The test for overall effect (pooled outcome) showed RE intervention had no effect on arterial stiffness (SMD = −0.09; 95% CI: −0.32, 0.13; *P* = 0.42), but risk of heterogeneity (*I*^2^) was 64%. Meta-regression results revealed a significant correlation (*P* = 0.042) between RE intensity and PWV changes. Consequently, the trials were subgrouped into high-intensity and low-to-moderate-intensity to identify the effective RE intensity. Subgroup analysis showed that low-to-moderate-intensity significantly decreased PWV (SMD = −0.34; 95% CI: −0.51, −0.17; *P* < 0.0001), while high-intensity had no effect (SMD = 0.24; 95% CI: −0.18, 0.67; *P* = 0.26). When trials separated into young and middle-aged, low-to-moderate-intensity notably decreased PWV in young (SMD = −0.41; 95% CI: −0.77, −0.04; *P* = 0.03) and middle-aged adults (SMD = −0.32; 95% CI: −0.51, −0.14; *P* = 0.0007), whereas high-intensity had no effect in both age groups.

**Conclusions:** Our findings demonstrated that RE intensity is the key variable in improving arterial stiffness. Low-to-moderate-intensity can prescribe as an effective non-pharmacological strategy to treat cardiovascular complications in young and middle-aged adults.

## Introduction

Arterial stiffness, measured from pulse wave velocity (PWV) is an independent risk factor for the development of cardiovascular disease (CVD). Increased arterial stiffness is closely associated with increased risk of morbidity and mortality in older populations and also in patients with chronic diseases (hypertension, type 2 diabetes, kidney disease, and stroke) (1–4). Arterial stiffening is represented by a gradual fragmentation and loss of elastin fibers, and accumulation of stiffer collagen fibers in the arterial wall (5). Several confounding factors, including aging, life style, diet, and concurrent disease are said to be involved in arterial stiffening and hypertension (6). Among various non-invasive and simplified protocols to measure the elastic properties of arteries, PWV is a widely recognized gold standard measure of arterial stiffness. Carotid-femoral PWV (cfPWV) and brachial-ankle PWV (baPWV) are the novel and most frequently used indices to determine arterial stiffness (7). The cfPWV is used to assess the central arterial stiffness, and baPWV is used to assess the whole-body arterial stiffness. Increased cfPWV and baPWV are the valid predictors of future incidence of CVD and mortality (1, 7). Given that, 1.0 m/s increase in cfPWV or baPWV can increase the risk of total cardiovascular events (12–14%), mortality (13–15%), and all-cause mortality (13–15%) (8). Therefore, reversing arterial stiffness (decreasing PWV) is a major achievement to prevent the development of hypertension and other clinical complications.

For decades, physical exercise, either resistance or aerobic, has been prescribed as a non-pharmacological intervention to promote overall health and to treat cardiovascular complications (9). Studies on exercise interventions are emerging due to the widespread benefits of exercise on human health (improving antioxidant status, lowering blood pressure, decreasing CVD risk factors, improving arterial stiffness, etc.). It has been stated that more time spent in physical activity is associated with lower arterial stiffness, whereas more time spent in sedentary behavior is associated with higher arterial stiffness (10). About the type, aerobic exercise has been confirmed to improve arterial stiffness in young, middle-aged, and older adults (11–13), as well as in patients with hypertension, metabolic syndrome, and diabetes (14–16).

However, the influence of RE on arterial stiffness or changes in PWV is still controversial. Some studies reported that RE training can improve the arterial stiffness in young healthy subjects (17, 18) and older hypertensive females (19). While others reported RE had no effect on arterial stiffness in young subjects (20) and individuals with metabolic syndrome (21). Contrary, RE training was reported to increase the arterial stiffness in healthy young subjects (22, 23), which decreases the vascular compliance. Besides, meta-analyses of research trials reported inconclusive results of RE intervention on changes in arterial stiffness. For instance, a meta-analysis reported increased arterial stiffness with high-intensity RE in young subjects, but moderate-intensity RE did not show such an association in middle-aged adults (24). Two recent meta-analyses based on the available evidence concluded that RE alone does not improve or impair the arterial stiffness in patients at risk for CVD (25) and in healthy individuals (26). It is worth noting that the included studies in these meta-analyses used different protocols to assess the arterial stiffness. Importantly, these meta-analyses reported moderate to high heterogeneity, and did not address the source of heterogeneity on exercise-induced changes in PWV.

High heterogeneity signifies the involvement of variables related to exercise protocol and/or patients' characteristics. In 2020, Ceciliato et al., who reported high heterogeneity without altering the PWVs, recommended further studies to identify the responsible variable of RE intervention (26). Typically, characteristics of RE (frequency, intensity, number of sets/repetitions, and duration) or participants (age, sex, and health status) are involved in the changes of arterial stiffness following intervention. Professional organizations like the American Heart Association (AHA) and American College of Sports Medicine (ACSM) also recommended practicing of RE training for further improvement of overall health and to overcome the practical limitations of aerobic exercise (27, 28). Therefore, we designed this study to systematically review and statistically analyze the impact of RE on arterial stiffness. We further aimed to identify the responsible RE variable that is involved in altering the PWV. Besides, the significance of RE “intensity” on improved arterial stiffness in young and middle-aged adults was emphasized based on the evidence from meta-regression and subgroup analyses.

## Methods

### Data Sources and Search Strategy

We used major electronic databases, including Web of Science, PubMed/MEDLINE, Google Scholar, EMBASE, Cochrane Library, ScienceDirect, CINAHL, Scopus, and Wiley Online Library for article search. The articles search was conducted until April 2021 using the main keywords: “resistance” or “strength” and “arterial stiffness.” In addition, “exercise” or “training” or “physical activity” should be in the title and abstract. The keywords “exercise,” “training,” or “physical activity” were independently used with “resistance” or “strength” and “arterial stiffness” and all searches were performed separately. In the search process, a filtering function of the databases was applied to filter the preliminary search results using “article,” “randomized controlled trial,” and “journals” options wherever applicable.

### Study Inclusion and Exclusion Criteria

The two authors (Y.Z. and Y.J.Z.) conducted the article search and selection independently. The author W.B.Y. provided additional review and insight. M.K. discussed and confirmed the disagreements on inclusion or exclusion of trials into the study. Initially, the titles and abstracts of searched articles were screened for relevance, and then the full text of the specified articles was obtained and carefully reviewed for the inclusion criteria. The following criteria were used to include the trials in this systematic review and meta-analysis: (1) studies were randomized controlled trials (RCTs) published in English; (2) resistance training is the only intervention in the trials, and is not combined with other interventions; (3) the control trial did not participate in any exercise, and maintained daily behavior or was sedentary; (4) participants were adults aged ≥18 years; (5) the duration of resistance exercise was 4 weeks or more; and (6) the outcome assessment was “arterial stiffness” measured as carotid-femoral PWV (cfPWV) or brachial-ankle PWV (baPWV), which is typically used to assess the central and whole-body arterial stiffness, respectively. We excluded studies according to these criteria: (1) non-randomized controlled trials or without control group; (2) combined with other interventions (aerobic exercise, vibration, supplementation, blood flow-restriction); (3) if participants were children or adolescents; (4) acute intervention study; (5) study did not report cfPWV or baPWV data; and (6) articles with repeated results, non-English, poor quality, or insufficient information about RE.

**Figure 1:**
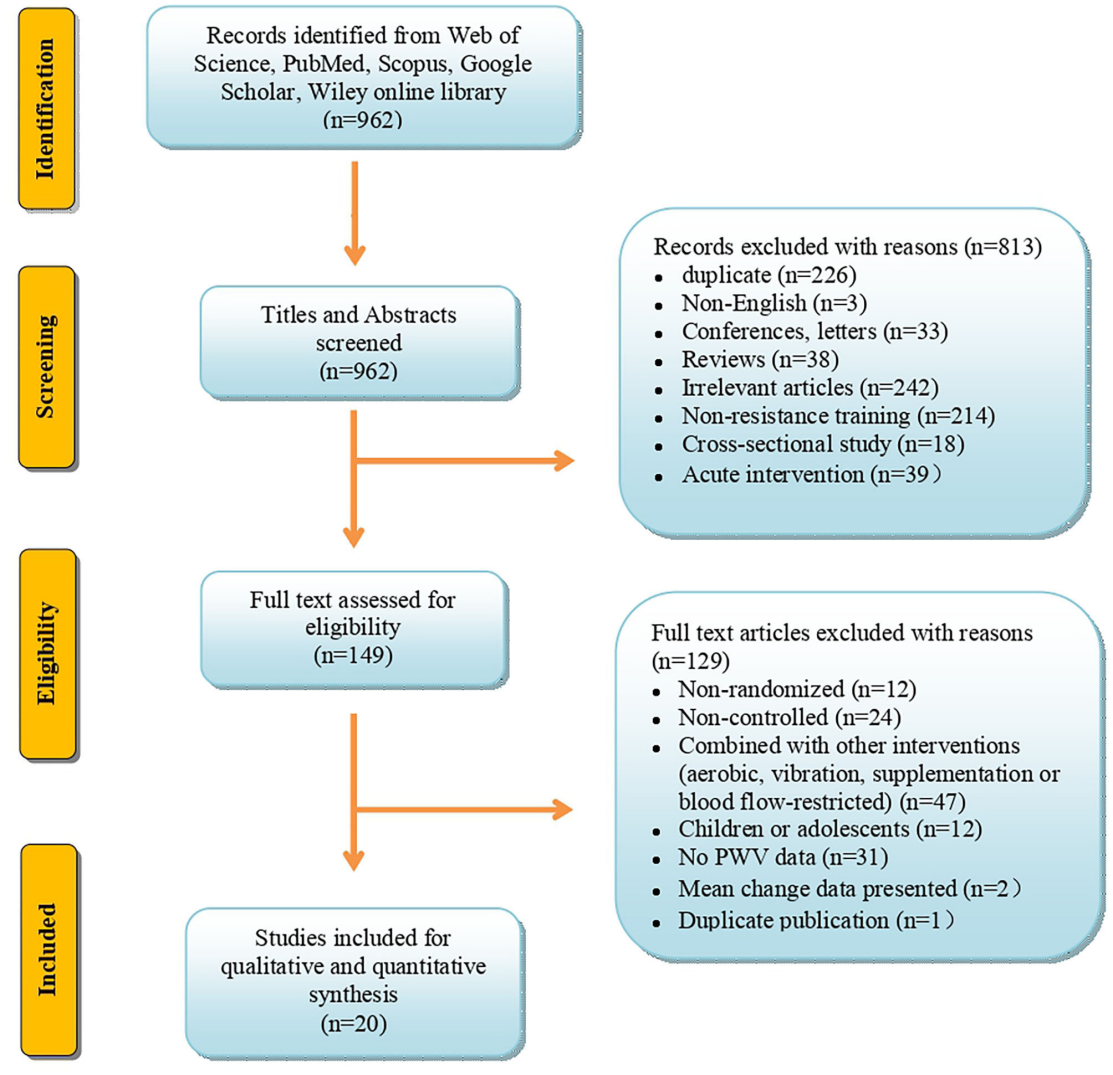
Flow diagram depicts the article search, exclusion, and inclusion according to the Preferred Reporting Items for Systematic Reviews and Meta-Analyses (PRISMA).

The specific details of the selection process, inclusion, and exclusion of articles for this study are presented in Figure 1. The article search and selection were performed according to the PRISMA (Preferred Reporting Items for Systematic Reviews and Meta-Analyses) guidelines as shown in Figure 1.

### Data Extraction From the Included Trials

The data from 20 eligible articles (30 trials), including basic information (authors, publishing year, and country in which the study was conducted), characteristics of participants (sex and age), resistance exercise protocols (intensity, repetitions, sets, frequency, and duration), and clinical outcomes (cfPWV and baPWV) were extracted and presented in Table 1. The data extraction was done by three independent review authors (Y.Z., Y.J.Z., and M.K.), and PWV was presented as mean and standard deviation (SD). If mean and SD were not available in the trials, we contacted the corresponding author for further information. If authors did not respond, standard errors were converted to SD, quartile data were converted to mean and SD (41), and data represented in tables were extracted to the nearest number by WebPlotDigitizer.

**Table 1:** Characteristics of the included studies.

**Study**	**Country**	**Age (Y)**	**Participants (M/F)**		**Description of RE**	**Intensity (%1RM)**	**Repetitions**	**Sets**	**Frequency(t/wk)**	**Duration (wk)**	**Outcome**
		**RE/Control**	**RE**	**Control**							
Au et al. (18)	Canada	23 ± 2/ 23 ± 2	16 (16/0)	14(14/0)	Leg press, seated row, bench press, cable hamstring curl, front planks, shoulder press, bicep curls, triceps extension, wide grip pull downs, and knee extension	75–90%	8–12	3	4	12	cfPWV
Au et al. (18)	Canada	23 ± 3/ 23 ± 2	16 (16/0)	14(14/0)	Leg press, seated row, bench press, cable hamstring curl, front planks, shoulder press, bicep curls, triceps extension, wide grip pull downs, and knee extension	30–50%	20–25	3	4	12	cfPWV
Beck et al. (29)	U.S.	21.1 ± 2.3/ 21.6 ± 3.1	15 (11/4)	15(10/5)	Leg extension, leg curl, leg press, lat pull down, chest press, overhead press, and biceps curl	60%	8–12	2	3	8	cfPWV
Cahu Rodrigues et al. (30)	Brazil	61 ± 8.25/ 59 ± 8	17 (6/11)	16(5/11)	Four sets of 2-min isometric contractions using a performed handgrip dynamometer	30%	nr	4	3	12	cfPWV
Casey et al. (20)	U.S.	21 ± 2.45/ 22 ± 2.97	24 (11/13)	18(8/10)	Leg extension, leg curl, leg press, lat pulldown, chest press, overhead press, and bicep curl	70%	8–12	2	3	12	cfPWV
Cortez-Cooper et al. (22)	U.S.	29 ± 1/ 27 ± 2	23 (0/23)	10(0/10)	Bench press, overhead press, weight-assisted parallel bar dip, dumbbell crossover pull, dumbbell rowing motion, latissimus dorsi pulldown, dumbbell curl, squat/leg press, high pull, deadlift, medicine ball drills, and abdominal exercises	75–85%	5–10	3–4	4	11	cfPWV
Croymans et al. (31)	U.S.	21.5 ± 2.34/ 21.85 ± 1.79	28 (28/0)	8(8/0)	Dumbbell (DB) squat, cable row, DB front lunge, DB row, barbell dead lift, DB triceps extension, DB bicep curl, DB step-up, barbell chest press, machine squat, DB overhead press, DB incline chest press, DB side raise, DB reverse fly, and abdominal crunches	70–85%	8–12	2–3	3	12	cfPWV
Devallance et al. (21)	U.S.	49 ± 12/ 44 ± 10.4	16 (4/12)	12(3/9)	Leg press, chest press, lat pull down, leg curl, shoulder press, and leg extension	70–85%	8–12	3	3	8	cfPWV
Devallance et al. (21)	U.S.	51 ± 11/ 51 ± 16	13 (4/9)	16(4/12)	Leg press, chest press, lat pull down, leg curl, shoulder press, and leg extension	70–85%	8–12	3	3	8	cfPWV
Greenwood et al. (32)	England	54.6 ± 10.6/ 49.5 ± 10.6	13 (7/6)	20(10/10)	Bench press, latissimus pulldown, bicep curl, triceps pull down, leg press, knee extension, hamstring curl, and calf raises	80%	8–10	3	3	12	cfPWV
Jaime et al. (33)	U.S.	64 ± 3.46/ 67 ± 2.83	12 (0/12)	8(0/8)	Leg press, leg extension, leg flexion, and calf raise	40%	15	2	3	12	baPWV cfPWV
Jones et al. (34)	New Zealand	55.8 ± 7.2/ 55.9 ± 7.1	26 (0/26)	25(0/25)	Leg press, leg extension, lying hamstring curl, machine bench press, lat pulldown, cable row, dumbbell shoulder press, dumbbell bicep curl, triceps pushdown, V-sit, abdominal crunches, and reverse abdominal crunches	60%	10–12	2–4	2	12	cfPWV
Miura et al. (35)	Japan	69.0 ± 6.5/ 68.9 ± 7.5	29 (0/29)	23(0/23)	Chest fly, biceps curl, push-up, bent-over row, upright row, overhead press, squat, front lunge, side lunge, straight-leg extension, heal raise, and outer thigh lift	50–60%	15–20	3–5	1	12	baPWV
Miura et al. (35)	Japan	69.5 ± 7.0/ 68.9 ± 7.5	25 (0/25)	23(0/23)	Chest fly, biceps curl, push-up, bent-over row, upright row, overhead press, squat, front lunge, side lunge, straight-leg extension, heal raise, and outer thigh lift	50–60%	15–20	3–5	2	12	baPWV
Miura et al. (19)	Japan	72.9 ± 5.7/ 69.7 ± 6.7	45 (0/45)	47(0/47)	Chest fly, biceps curl, push-up, bent-over row, upright row, overhead press, squat, front lunge, side lunge, straight-leg extension, heal raise, and outer thigh lift	50–60%	15–20	3–5	2	12	baPWV
Miura et al. (19)	Japan	72.0 ± 7.1/ 71.8 ± 5.6	55 (0/55)	53(0/53)	Chest fly, biceps curl, push-up, bent-over row, upright row, overhead press, squat, front lunge, side lunge, straight-leg extension, heal raise, and outer thigh lift	50–60%	15–20	3–5	2	12	baPWV
Okamoto et al. (36)	Japan	18.9 ± 0.3/ 19.9 ± 1.2	10 (0/10)	9(0/9)	Arm curl (2s eccentric phase)	80%	10	5	3	8	baPWV
Okamoto et al. (36)	Japan	19.1 ± 0.3/ 19.9 ± 1.2	10 (0/10)	9(0/9)	Arm curl (2s concentric phase)	80%	10	5	3	8	baPWV
Okamoto et al. (37)	Japan	19.4 ± 0.2/ 19.4 ± 0.2	10 (10/0)	9(9/0)	Chest press, arm curl, lateral pull down, seated row, shoulder press, leg extension, leg curl, leg press, and sit-up (3-s lowering phase and 3-s lifting phase)	40%	10	5	2	8	baPWV
Okamoto et al. (23)	Japan	20.2 ± 0.4/ 20.1 ± 0.3	10 (7/3)	10(6/4)	Chest presses, arm curls, seated rows, shoulder presses, and lat pull downs	80%	8–10	5	2	10	baPWV
Okamoto et al. (23)	Japan	20.0 ± 0.5/ 20.1 ± 0.3	10 (7/3)	10(6/4)	Leg presses, squats, seated calf raises, leg extensions, and leg curls	80%	8–10	5	2	10	baPWV
Okamoto et al. (23)	Japan	19.6 ± 1.26/ 19.7 ± 0.95	10 (10/0)	10(10/0)	Chest presses, arm curls, seated rowing, leg curls, leg presses, and sit-ups (1-s lifting phase and 3-s lowering phase)	80%	8–10	5	2	10	baPWV
Okamoto et al. (23)	Japan	19.2 ± 0.95/ 19.7 ± 0.95	10 (10/0)	10(10/0)	Chest presses, arm curls, seated rowing, leg curls, leg presses, and sit-ups (3-s lifting phase and 1-s lowering phase)	80%	8–10	5	2	10	baPWV
Okamoto et al. (17)	Japan	18.5 ± 0.5/ 18.6 ± 0.5	13 (10/3)	13(9/4)	Chest press, arm curl, seated row, lateral pull down, leg press, leg extension, leg curls, and sit-ups	50%	10	5	2	10	baPWV
Okamoto et al. (38)	Japan	19.3 ± 0.7/ 19.1 ± 0.6	10 (5/5)	10(5/5)	Chest presses, arm curls, seated rowing, leg curls, and leg presses	50/80%	10	2/3	2	10	cfPWV
Okamoto et al. (38)	Japan	19.1 ± 0.7/ 19.1 ± 0.6	10 (5/5)	10(5/5)	Chest presses, arm curls, seated rowing, leg curls, and leg presses	80/50%	10	3/2	2	10	cfPWV
Werner et al. (39)	U.S.	22.9 ± 2.9/ 21.2 ± 2.8	10 (10/0)	10(10/0)	Back squats, flat bench press, seated rows, shoulder press, bicep curls, triceps extension, standing calf raises, seated leg curls, and seated leg extension	80–90%	3–8	2–3	3–5	12	cfPWV
Werner et al. (39)	U.S.	20.9 ± 3.2/ 21.2 ± 2.8	10 (10/0)	10(10/0)	Back squats, flat bench press, seated rows, shoulder press, bicep curls, triceps extension, standing calf raises, seated leg curls, and seated leg extension	50–70%	10–15	3–4	3–5	12	cfPWV
Yoshizawa et al. (40)	Japan	47 ± 6.63/ 49 ± 10.39	11 (0/11)	12(0/12)	Leg curl, leg press, hip adduction, hip flexion, vertical press, and sit-ups	60%	10	3	2	12	cfPWV

*Y, years; M/F, male/female; RE, resistance exercise; 1RM, one-repetition maximum; t/wk, times/week; cfPWV, carotid-femoral pulse wave velocity; baPWV, brachial-ankle PWV; nr, not reported*.

### Risk of Bias Assessment

The risk of bias for the included articles was determined according to the Cochrane Collaboration tool (42). Two of the three review authors (Y.Z., Y.J.Z., or W.B.Y.) independently assessed the risk of bias, and possible discrepancies were resolved by discussing with the other review author (M.K.). The source of bias, such as selection bias (random sequence generation and allocation concealment), performance bias (blinding of participants and personnel), detection bias (blinding of outcome assessment), attrition bias (incomplete outcome data), reporting bias (selective reporting), and other bias were detected. The detailed judgment of the risk of bias of included trials is summarized in the Results section.

### Subgroup Division and Observed Outcomes

The intensity of RE performed by individuals in each trial was converted and presented as 1-RM percentages (43, 44). Based on the intensity, included trials were categorized into two subgroups, including low-to-moderate-intensity and high-intensity trials. The intensity between 30 and 70% 1-RM is considered as low-to-moderate-intensity RE, and intensity between 70 and 100% 1-RM is considered as high-intensity RE. This subgroup category was followed according to the ACSM Guidelines for Exercise Testing and Prescription (28). When two or more different intensities were used in training, the average intensity was used to classify into low-to-moderate-intensity or high-intensity RE.

As a gold standard approach for assessing the arterial stiffness, the outcome values of cfPWV (used to assess central arterial stiffness) and baPWV (used to assess whole-body arterial stiffness) were included in the meta-analysis.

### Statistical Analyses

The data analysis was performed using statistical software of the Cochrane Collaboration Review Manager (RevMan, version 5.3, Copenhagen, Denmark). The main statistical procedures were heterogeneity analysis, computation, and verification of combined effect size. The fixed effect model was used for meta-analysis, if no significant difference was found in heterogeneity analysis (*p* > 0.05). The random effect model was used, if heterogeneity was found significant (*p* < 0.05). We used STATA version 12 (StataCorp, College Station, TX) for the analyses of sensitivity, publication bias, and meta-regression. The changes in pulse wave velocity after exercise were found to be correlated with the intensity variable (Coef. = 0.382, T = 2.13, *p* = 0.042), and not with other variables (intervention duration, frequency, sets, and repetitions). Hence, trials were categorized into two subgroups, high-intensity (70–100%, 1-RM) and low-to-moderate-intensity (30–70%, 1-RM) to identify the effective intensity of RE (28). The differences between the subgroups were also analyzed, and indicated as a significant difference.

Upon the heterogeneity significance (pooled outcome), we performed another subgroup analysis to examine the association between age and RE intensities on the outcomes (cfPWV and baPWV). According to the participants' age, trials were subgrouped into young (<40 years) and middle-aged (≥40 years) (24), and the influence of RE intensities on outcome changes was analyzed in both age groups. Taking into account the differences of outcomes in the studies and the evaluation of the effect size, the standardized mean difference (SMD) was used to determine the magnitude of the RE effect, where the value <0.2 was defined as trivial, 0.2–0.3 as small, 0.4–0.8 as moderate, and >0.8 as large (45). The SMD was expressed as 95% confidence interval (CI). The statistical heterogeneity across different trials in the meta-analysis was assessed by the *I*^2^ statistic, where <25% indicates a low risk of heterogeneity, 25–75% indicates a moderate risk of heterogeneity, and >75% indicates a considerable risk of heterogeneity (46).

## Results

### Search Results and Article Selection

We identified a total of 962 articles from the electronic databases, Web of Science, PubMed/MEDLINE, Scopus, ScienceDirect, EMBASE, Cochrane Library, CINAHL, Google Scholar, and Wiley Online Library. After screening the titles and abstracts, 813 articles were excluded, and the remaining 149 were selected for the full-text assessment. Of these, 129 articles were further excluded with reasons explained in Figure 1. Finally, 20 articles met the inclusion criteria, and were included in the systematic review and meta-analysis. The informative flow-chart of article search and selection according to PRISMA guidelines was summarized in Figure 1.

### Description of the Included Articles

The included trials in this systematic review and meta-analysis (*n* = 20) were intercontinental, published between 2005 and 2020. A majority of the articles (*n* = 9) were conducted in Japan (17, 19, 23, 35–38, 40), followed by the U.S. (*n* = 7) (20–22, 29, 31, 33, 39) and each one from Canada (18), Brazil (30), England (32), and New Zealand (34). Of these, five studies recruited only male participants, seven studies recruited only females, and eight studies recruited a combination of both males and females. The number of participants in the RE group ranged from 10 to 55, and the number of participants in the control group ranged from 9 to 53. The total sample size was 519 (187 males and 332 females) in the RE trial, and 462 (156 males and 306 females) in control trial. The age of participants ranged from 18 to 88 years old. The duration of RE intervention was between 8 and 12 weeks with a frequency of 1–5 times per week. The intensity of RE (%1-RM) ranged from 30 to 90% 1-RM, and sets of repetitions ranged from 3 to 25. The characteristics of participants and RE intervention along with publication details were summarized in Table 1.

### Summary of the Risk of Bias

**Figure 2:**
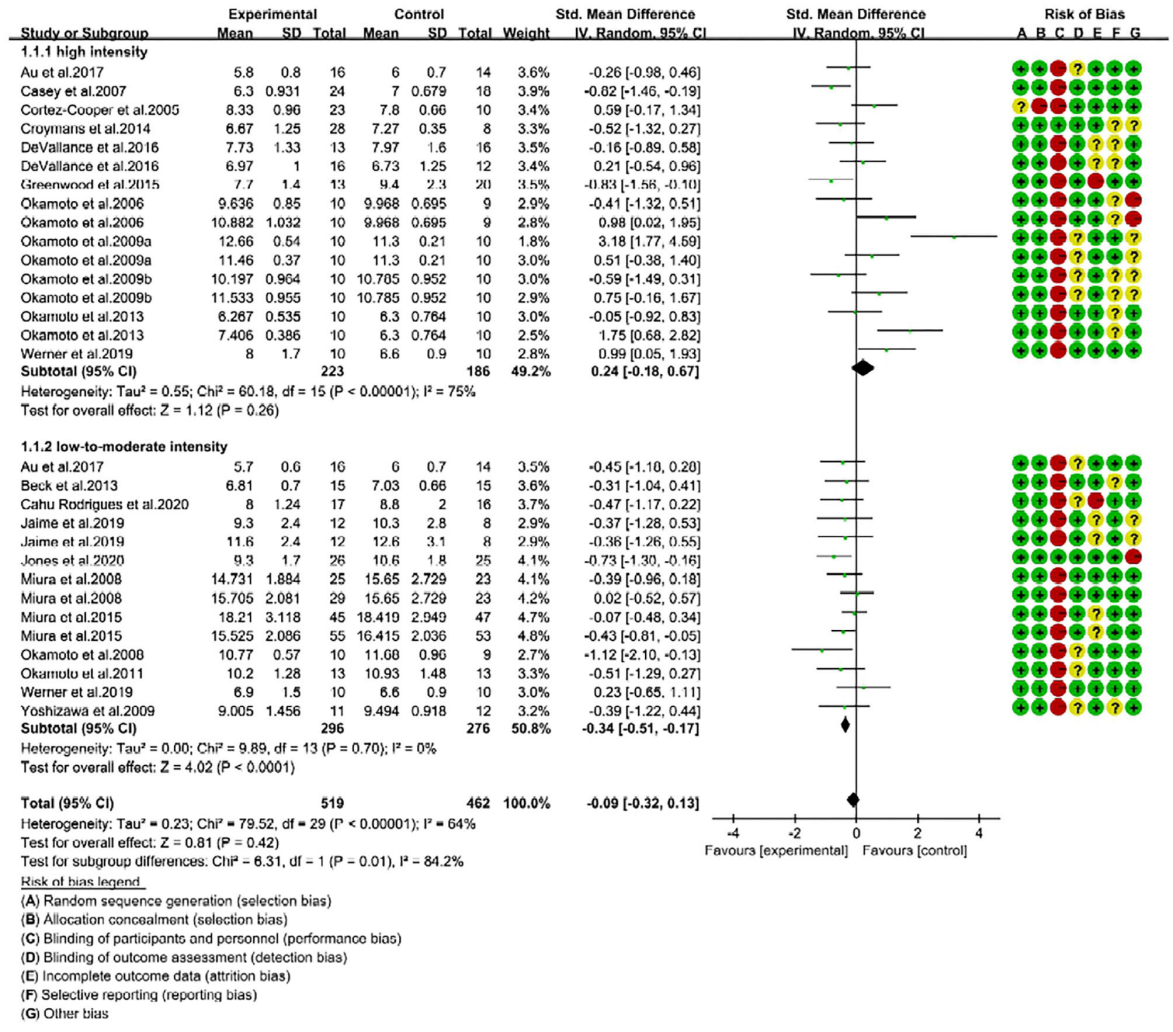
Forest plot of pulse wave velocity (PWV) changes with different intensities of RE in adults (m/s). SD, standard deviation; IV, inverse variation; CI, confidence interval; df, degrees of freedom. Risk of bias: green (+) indicates low risk of bias; red (–) high risk of bias; and yellow (?) unclear risk of bias.

The Cochrane Collaboration method was employed to assess the risk of bias for the included trials, and the detailed statement was presented in Figure 2. For the selection bias, all the trials except one (22) were randomly assigned, and five trials reported the methods used for randomization (30–34). For the performance bias, except two trials (31, 34), all trials were judged to have high risk of bias for blinding participants to an exercise intervention. In those two studies (31, 34) all participants and authors (except research coordinator) were blinded to the recruitment, randomization, and experiment execution. Typically it is not possible to blind the participants in an exercise intervention, and reporting such a high risk of bias does not mean it influences or compromises the quality of the study (47, 48). Instead, other variables, including the level of study attrition, poor intervention adherence, and selective reporting bias are the most common issues around the high risk of bias that would impact quality of the study (49). In our assessment, two studies reported to have attrition bias because of the high attrition rate (30, 32). In addition, two trials were identified with other risks of bias (34, 36), one conducted a circuit resistance training mixed with a small amount of aerobic exercise components (34), and the other one conducted local training limited to only arm curls on the left side (36).

The sensitivity analyses results showed that no trial had a significant impact on the total effect size. However, the funnel plot and Egger linear regression test (Egger's test) (*t* = 2.52; *p* = 0.018; 95% CI: 0.46, 4.46) showed a publication bias (Supplementary Figure 1). It can be seen from the funnel plot that one trail had relatively large bias (23), but when it was not considered, the publication bias was eliminated (*t* = 1.75; *p* = 0.092; 95% CI: −0.29, 3.57). This trial reported significantly increased baPWV (SMD = 3.18, 95% CI: 1.77, 4.59) with upper limb resistance training.

### Influence of Resistance Exercise Intervention on Arterial Stiffness

Arterial stiffness is determined by monitoring the changes of cfPWV and baPWV in adults (7). In our meta-analysis (*n* = 30), 17 trials reported cfPWV data and 13 reported baPWV data. We combined the effect size of two outcomes (cfPWV and baPWV) under the random effects model, and used SMD to determine the effect size of RE intervention on arterial stiffness. The pooled results showed that RE intervention had no effect on PWV in adults (SMD = −0.09; 95% CI: −0.32, 0.13). The overall effect of RE on arterial stiffness was not statistically significant (*P* = 0.42), but heterogeneity was moderate (*I*^2^ = 64%; Supplementary Figure 2). These results direct us to find out the source of heterogeneity.

### RE Intensity Is Associated With Reduction of PWV

We performed meta-regression analysis to determine the influence of RE variables, such as frequency, intensity, duration, sets, and repetitions on PWV changes. The results revealed that only the “intensity” variable is significantly correlated with the changes of PWV after RE intervention (Coef. = 0.382, *t* = 2.13, *P* = 0.042), while frequency (Coef. = 0.007, *t* = 0.04, *P* = 0.969), duration (Coef. = −0.095, *t* = −1.10, *P* = 0.282), sets (Coef. = 0.126, *t* = 0.94, *P* = 0.354), and repetition (Coef. = −0.055, *t* = −1.38, *P* = 0.179) were not correlated with the changes of PWV (Table 2).

**Table 2:** Meta-regression analysis for the changes in pulse wave velocity (PWV, m/s) and resistance exercise (RE) variables.

**RE variables**	**Coefficient**	**Standard error**	***T* value**	***P*-value**	**[95% Conf. interval]**	
Intensity	0.382	0.179	2.13	0.042^*^	0.015	0.749
Frequency	0.007	0.175	0.04	0.969	−0.352	0.366
Duration	−0.095	0.087	−1.10	0.282	−0.273	0.083
Sets	0.126	0.134	0.94	0.354	−0.148	0.401
Repetitions	−0.055	0.040	−1.38	0.179	−0.136	0.027

* *Represents a significant correlation between PWV change and RE variables*.

Based on intensity, we then categorized the trials into two subgroups, namely high-intensity (70–100% 1-RM, 16 trials) and low-to-moderate-intensity (30–70% 1-RM, 14 trials). Consequently, we determined the effectiveness of two different RE intensities on reduction of arterial stiffness in adults.

### Low-to-Moderate-Intensity Effectively Reduces PWV Than High-Intensity RE

We performed subgroup analysis to identify the effective RE intensity that could improve arterial stiffness in adults. The subgroup analysis results showed that the changes in PWV were not significant after high-intensity RE (SMD = 0.24; 95% CI: −0.18, 0.67; *P* = 0.26), which indicates high-intensity RE was unable to improve the arterial stiffness (Figure 2). Interestingly, we noticed that low-to-moderate-intensity RE significantly improved arterial stiffness in adults. This was evidenced by a significant reduction of PWV in RE trials (SMD = −0.34, 95% CI: −0.51, −0.17; *P* < 0.0001) with no risk of heterogeneity (*I*^2^ = 0%). From the subgroup analysis, we further noticed that there is a significant difference (*P* = 0.01) in the effect size of RE on arterial stiffness (SMD changes) between high- and low-to-moderate intensity trials (Chi^2^ = 6.31, *I*^2^ = 84.2%) (Figure 2).

### RE Improves Arterial Stiffness in Middle-Aged Adults

**Figure 3:**
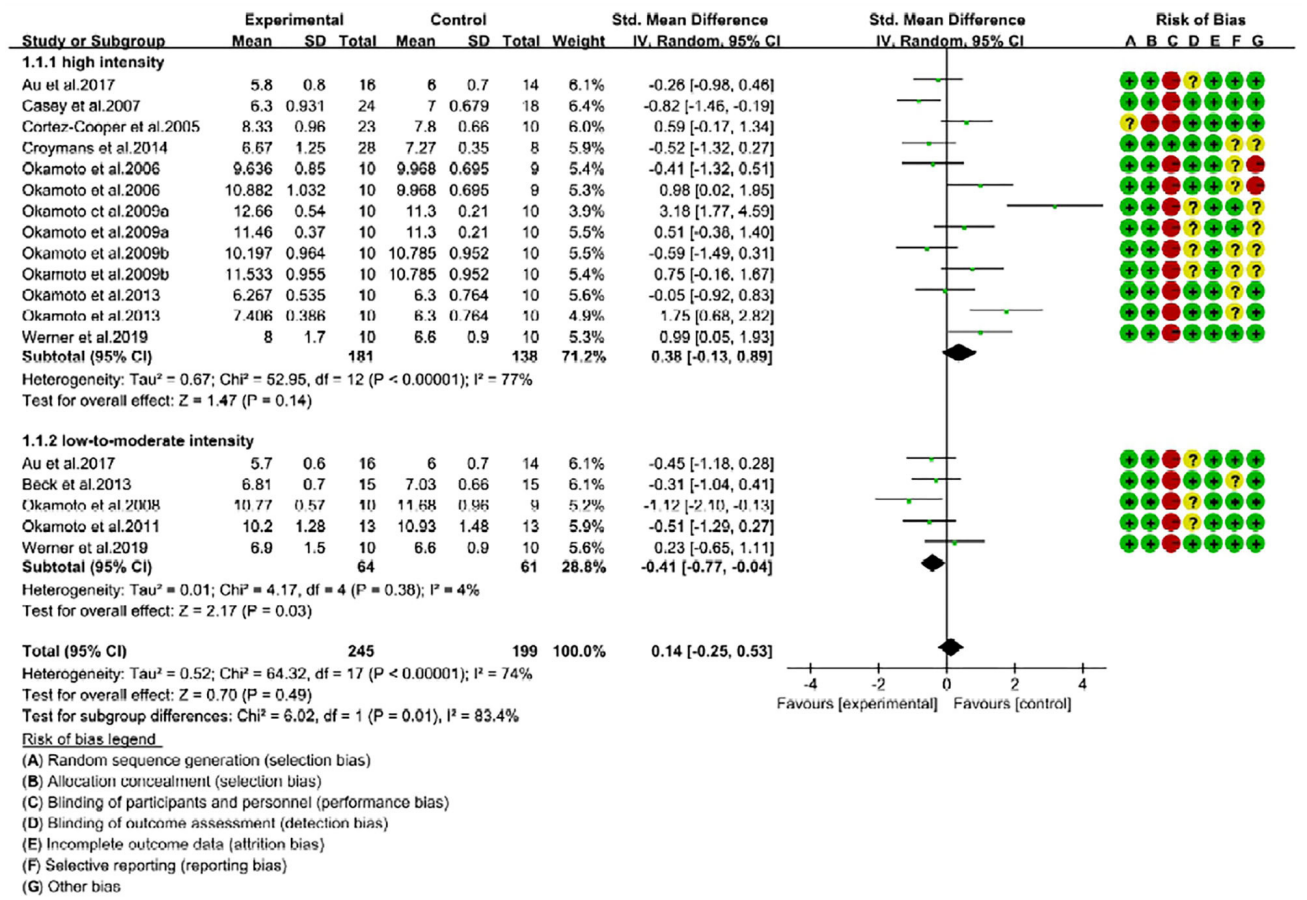
Forest plot of pulse wave velocity (PWV) changes with different intensities of RE in young individuals (m/s). SD, standard deviation; IV, inverse variation; CI, confidence interval; df, degrees of freedom. Risk of bias: green (+) indicates low risk of bias; red (–) high risk of bias; and yellow (?) unclear risk of bias.

**Figure 4:**
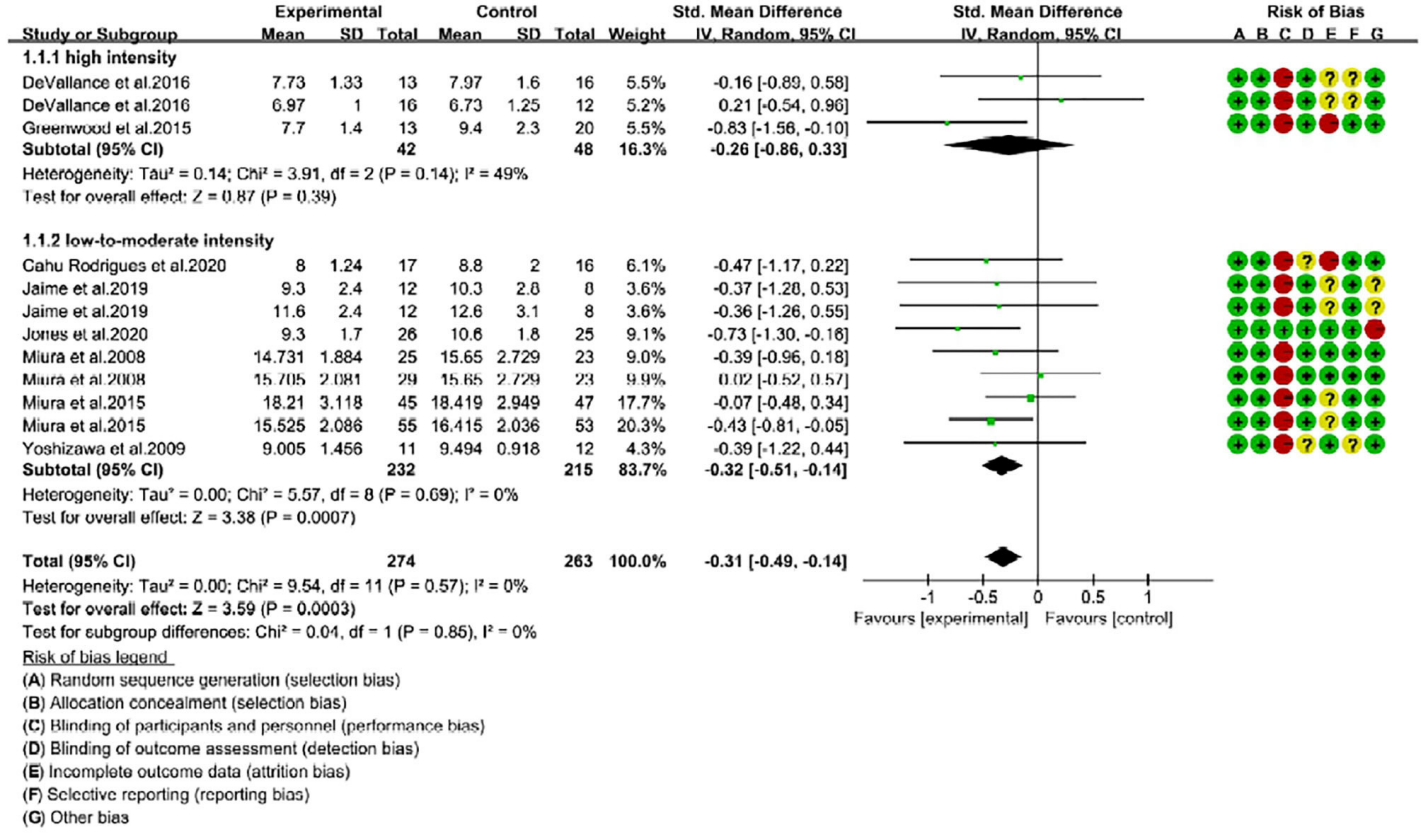
Forest plot of pulse wave velocity (PWV) changes with different intensities of RE in middle-aged adults (m/s). SD, standard deviation; IV, inverse variation; CI, confidence interval; df, degrees of freedom. Risk of bias: green (+) indicates low risk of bias; red (–) high risk of bias; and yellow (?) unclear risk of bias.

We next hypothesized that the beneficial effects of RE on arterial stiffness could be influenced by the “age” of the individuals. To explore this phenomenon, we categorized the trials into young (<40 years, 18 trials) and middle-age or old groups (≥40 years, 12 trials) (24). Regardless of intensity, the overall RE intervention had no effect on PWV changes in young participants (SMD = 0.14; 95% CI: −0.25, 0.53; *P* = 0.49, Figure 3). Nevertheless, as reported in Figure 4, middle-aged adults showed positive response to RE intervention, and the decreased PWV was statistically significant (SMD = −0.31; 95% CI: −0.49, −0.14; *P* = 0.0003) with no risk of heterogeneity (*I*^2^ = 0%). These findings indicate that RE improved arterial stiffness in middle-aged adults, but not in young individuals.

### Association Between RE Intensities and Age on Arterial Stiffness

We performed another subgroup analysis to reveal the association between RE intensities and age on improvement of arterial stiffness. For the young individuals, high-intensity RE (13 trials) had no effect on PWV changes (SMD = 0.38; 95% CI: −0.13, 0.89; *P* = 0.14), and trials indicated considerable risk of heterogeneity (*I*^2^ = 77%). In contrast, low-to-moderate-intensity (5 trials) resulted in a significant (*P* = 0.03) reduction of PWV in young adults (SMD = −0.41; 95% CI: −0.77, −0.04) with low risk of heterogeneity (*I*^2^ = 4%). Furthermore, the test for subgroup difference between low-to-moderate-intensity and high-intensity was significantly different (Chi^2^ = 6.02; *P* = 0.01; *I*^2^ = 83.4%). These results witnessed that only low-to-moderate-intensity improved the arterial stiffness in young individuals (Figure 3).

Next, we found that middle-aged adults were not positively responded to high-intensity RE, as reported insignificant changes of PWV after intervention (SMD = −0.26; 95% CI: −0.86, 0.33; *P* = 0.39) (Figure 4). Noteworthy, middle-aged adults following low-to-moderate-intensity were represented with a significant improvement in arterial stiffness. The PWV mean change in low-to-moderate-intensity trials was extremely significant (*P* = 0.0007) with SMD of −0.32, 95% CI is −0.51 to −0.14, and low risk of heterogeneity (*I*^2^ = 0%). Subgroup analysis showed no significant difference (*P* = 0.85) between high- and low-to-moderate intensity trials (Figure 4). Taken together, our analysis revealed that RE with low-to-moderate-intensity promoted arterial stiffness in young and middle-aged adults, while high-intensity is ineffective in both age groups.

## Discussion

To the best of our knowledge, this is the first systematic review and meta-analysis to demonstrate the influential role of RE intensities on arterial stiffness in young and middle-aged adults. Arterial stiffness is a growing global-health burden associated with increased risk of cardiovascular events, hypertension, dementia, and mortality. However, reversing arterial stiffness or decreasing PWV (m/s) could prevent the incidence of such diseases (4, 6, 8). Here we determined the changes of PWV (a gold standard measure of arterial stiffness) in adults, who participated in RE intervention at least for 8 weeks. Meta-analysis results showed RE (irrespective of intensity) had no effect on arterial stiffness, but risk of heterogeneity was moderate. Meta-regression analysis revealed that RE intensity is correlated with decreased PWV, which indicates “intensity” is the key variable in improving arterial stiffness. Subgroup analysis showed decreased PWV is effective (−0.34) with low-to-moderate-intensity. Conversely, high-intensity RE is ineffective to decrease the PWV. About the age factor, we further reported low-to-moderate-intensity decreased PWV in young and middle-aged adults, while high-intensity is unable to improve the arterial stiffness in either age group. These findings provided evidence that RE interventions with low-to-moderate-intensity can reverse the arterial stiffness, and thereby prevent the occurrence and/or progression of cardiovascular events. Our meta-regression and subgroup analyses are the newly added evidence to the existing review that summarizes RE intensity effect on arterial stiffness without statistical analysis (50).

It is well-documented that arterial stiffness determined by increased PWV is associated with age and systolic blood pressure, and is an independent risk factor for CVD and mortality (7, 8, 51). The included trials in our study addressed the effect of resistance training on arterial stiffness of young and middle-aged adults by reporting the changes in cfPWV and/or baPWV. The test for overall effect (pooled outcome) showed RE intervention for a period of 8–12 weeks did not influence the arterial stiffness in adults. Similar to our findings, two recent meta-analyses concluded that resistance training had no effect on arterial stiffness in persons with high risk of CVD (25) and also in healthy individuals (26). However, the source of heterogeneity (*I*^2^) that is 40% in Evans' study (25) (5 trials) and 86% in the study by Ceciliato et al. (26) (15 trials) was not addressed clearly. The moderate heterogeneity (*I*^2^ = 64%) reported in our analysis suggests the possible involvement of exercise variables on PWV changes. Through meta-regression analysis, we found exercise intensity is associated with reduction of PWV rather than exercise frequency, duration, sets, and repetitions. To be particular, low-to-moderate-intensity had a greater beneficial effect in improving the arterial stiffness, while high-intensity was ineffective to do so.

Our findings are quite interesting and are different from the previous reviews and meta-analyses, which investigated RE intensities effect on arterial stiffness. For instance, a meta-analysis by Miyachi demonstrated that high-intensity RE was associated with increased arterial stiffness, but moderate-intensity RE did not show such association in adults (24). In contrast, another meta-analysis showed RE had no effect (positive or negative) on PWV, and there was no association between RE intensity and arterial stiffness (52). A recent systematic review emphasized that 4-week RE intervention with a frequency of 2 times per day per week may decrease arterial stiffness. Nevertheless, the influence of intensity on arterial stiffness was not addressed in this review (53). To address the RE intensity effect, a review by Figueroa et al. stated that low- and high-intensity resistance training may not influence arterial stiffness. Irrespective of intensity, the overall RE may decrease central and peripheral blood pressure in middle-aged and older adults with elevated blood pressure at baseline (50). However, these two reviews (50, 53) did not explore the correlations between PWV and exercise characteristics, and did not provide any statistical or meta-analysis evidence to convince their conclusions. In our study, through meta-regression and subgroup analyses, we provided evidence that low-to-moderate-intensity RE effectively improved arterial stiffness in young and middle-aged adults.

Regardless of intensity, we found RE intervention (the overall effect) significantly decreased PWV in middle-aged adults, but not in young individuals. However, when trials were subgrouped based on the intensity, only low-to-moderate-intensity improved arterial stiffness in young and middle-aged adults, while high-intensity had no effect in both age groups. These findings opened for debate why high-intensity RE is not beneficial to improve arterial stiffness in individuals separated into similar age groups. Relatively with a lesser number of trials (4), Miyachi (2013) reported significantly increased arterial stiffness in young subjects after high-intensity RE, while moderate-intensity (3 trials) had no effect on middle-aged adults. This tendency might be due to the lower baseline values in young adults, and higher arterial stiffness in middle-aged adults (24). It is further suggested that low-intensity may decrease the systemic arterial stiffness (baPWV) in young healthy adults or not influence the arterial stiffness in middle-age and older adults (50). The latest systematic review stated that acute low-intensity RE with blood flow restriction intervention (2 articles) had positive or negative effects on arterial stiffness in healthy young adults, while chronic intervention (3 articles) had neutral effects on healthy young and older adults (54). In middle-aged women, moderate-intensity RE (60%, 1-RM) for 12 weeks did not produce any unfavorable effects on vasculature, as revealed by unchanged cfPWV and femoral-ankle PWV; however, muscle strength was increased (40). A study conducted on young healthy men reported increased arterial stiffness (cfPWV) and aortic augmentation index (Aix) following acute RE program (60%, 1-RM), and advised future studies to examine the long-term effect of RE on arterial stiffness (55). A few years later, another study on young and older women represented with unchanged cfPWV or femoral-tibialis posterior arterial stiffness after 8 weeks of high-intensity resistance training (3-time/week, ~80% 1-RM) (56). These equivocal conclusions from reviews and research trials may be due to the variances in article inclusion criteria, RE protocols (frequency, intensity, sets, repetitions, duration), and/or subjects' characteristics (age, sex, bodyweight, health status).

### Mechanism and Factors Involved in Regulation of Arterial Stiffness

The detailed mechanism for the diverse effect of RE on arterial stiffness has yet to be fully elucidated. We postulated that internal factors, including muscle tone, sympathetic nerve activity, blood pressure, blood circulation, and endothelial function could influence the arterial stiffness following exercise intervention. Okamoto and team demonstrated that upper- but not lower-limb high-intensity RE increased arterial stiffness (baPWV) in young adults. This was accompanied by an increased plasma norepinephrine concentration, which reflects sympathetic nervous system activity. The activation of the sympathetic system may acutely affect the arterial distensibility through complex interactions between large arterial smooth muscle tone and distending blood pressure (23). The central arterial function is influenced by endothelial function. The key vasoactive agents, nitric oxide (NO) and endothelin-1 (ET-1) produced by endothelial cells can alter the smooth muscle tone, and thereby regulate large artery stiffness (57). On the other hand, decreased conduit artery endothelial function is associated with increased peripheral artery PWV and central pulse pressure (58).

Decreased central blood pressure and peripheral PWV (not cfPWV) in pre-hypertensive patients after whole-body resistance training (60%, 1RM, 8 weeks) are associated with improved endothelial function and vasoactive substances (29). It is further disclosed that RE reduced blood pressure and improved brachial artery FMD (flow-mediated dilation) in young pre-hypertensive patients with concurrently increased NO bioavailability and decreased circulating ET-1 (29). Higher ET-1 production is associated with increased arterial stiffness in young strength-trained men (weight lifters), while plasma NO concentrations remain unchanged (59). Improved arterial stiffness in obese adolescent girls after RE plus aerobic exercise intervention was represented by an increased plasma NO level and unchanged ET-1 (60). Besides, aerobic exercise combined with low-intensity RE reported to increase basal NO production, and decrease arterial stiffness without changing the bodyweight in healthy older adults (61). Improved endothelial NO-mediated vasodilatory function may result in decreased PWV (50). Literature revealed that high-intensity RE may not produce favorable effects on endothelial function in healthy men (23, 62). High-intensity RE may increase the blood pressure acutely and sympathetic activity chronically, which contribute to an increase in arterial stiffness. Besides, moderate- or high-intensity RE is favorable on brachial artery or forearm endothelial function in overweight postmenopausal women (63, 64) and middle-aged adults (65) with elevated blood pressure. Interestingly, low-intensity RE with slow lifting and lowering, and short inter-set rest periods showed positive effects on endothelial function in healthy adults (17, 37). Taken together, high-intensity RE can increase the sympathetic nerve activity, muscle tone, blood pressure, and circulation resistance. Such elevations eventually lead to deleterious adaptation of vascular smooth muscle, and thereby increase arterial stiffness. On the other hand, low-intensity RE may not increase sympathetic nerve activity or muscle tone. The proper muscle contraction with low-intensity also promotes blood circulation and thereby improves vascular endothelial function and arterial stiffness.

## Limitations

In our analyses, we mixed the trials that investigated the effect of RE in healthy individuals as well as patients. Although our results showed that low-to-moderate intensity RE is beneficial in improving the arterial stiffness in young and middle-aged adults, the beneficial effects of RE intensity in specific population, like hypertensive or diabetic patients, remains uncertain. The small sample size in the included trials (not many) might be a limitation for those studies. Very small sample size can undermine the internal and external validity of the study, and it is hard to determine whether the changes in outcome measures are true or statistically different. Further analyses with large-scale sample size are required to confirm negative or neutral effects of high-intensity RE on the arterial stiffness of an aged population with or without the existence of chronic diseases.

## Conclusions

The evidence from our systematic review, meta-analysis, and meta-regression analysis confirmed that RE intensity is the key variable to promote arterial stiffness. Precisely, low-to-moderate-intensity RE is effective in improving arterial stiffness in young and middle-aged adults. In contrast, high-intensity RE is ineffective in decreasing the PWV. Therefore, practicing high-intensity RE should be cautious in particular age groups/patients due to the unfavorable effects on arterial stiffness. Practicing of low-to-moderate-intensity RE is beneficial to promote arterial stiffness that may aid to reduce the risk for cardiovascular diseases.

## Data Availability Statement

The original contributions presented in the study are included in the article/Supplementary Material, further inquiries can be directed to the corresponding authors.

## Author Contributions

YZ, Y-JZ, and MK designed the study, performed article search and screening, reviewed the full-text articles, and extracted the data. YZ and Y-JZ performed statistical analyses and drafted the manuscript. WY provided additional suggestions and assisted in interpretation of data. YZ and MK revised and finalized the manuscript. All authors have read and approved the submission.

## Funding

This study was supported by the grant from the National Social Science Foundation of China (Grant Number 17BTY008), China.

## Conflict of Interest

The authors declare that the research was conducted in the absence of any commercial or financial relationships that could be construed as a potential conflict of interest.

## Publisher's Note

All claims expressed in this article are solely those of the authors and do not necessarily represent those of their affiliated organizations, or those of the publisher, the editors and the reviewers. Any product that may be evaluated in this article, or claim that may be made by its manufacturer, is not guaranteed or endorsed by the publisher.
