# Supplementary material for: Low-to-Moderate-Intensity Resistance Exercise Effectively Improves Arterial Stiffness in Adults: Evidence From Systematic Review, Meta-Analysis, and Meta-Regression Analysis
Source: Front Cardiovasc Med. 2021 Oct 11;8:738489. doi: 10.3389/fcvm.2021.738489 (PMC8544752; doi:10.3389/fcvm.2021.738489)

**Figure 1S.** Funnels plots (Egger’s test) for the sensitivity analysis of the included trials.

**Figure 2S.** Forest plot of pulse wave velocity (PWV) changes with RE intervention in adults (m/s). SD, standard deviation; IV, inverse variation; CI, confidence internal; df, degrees of freedom. Risk of bias: green (+) indicates low risk of bias; red (−) high risk of bias and yellow (?) unclear risk of bias.


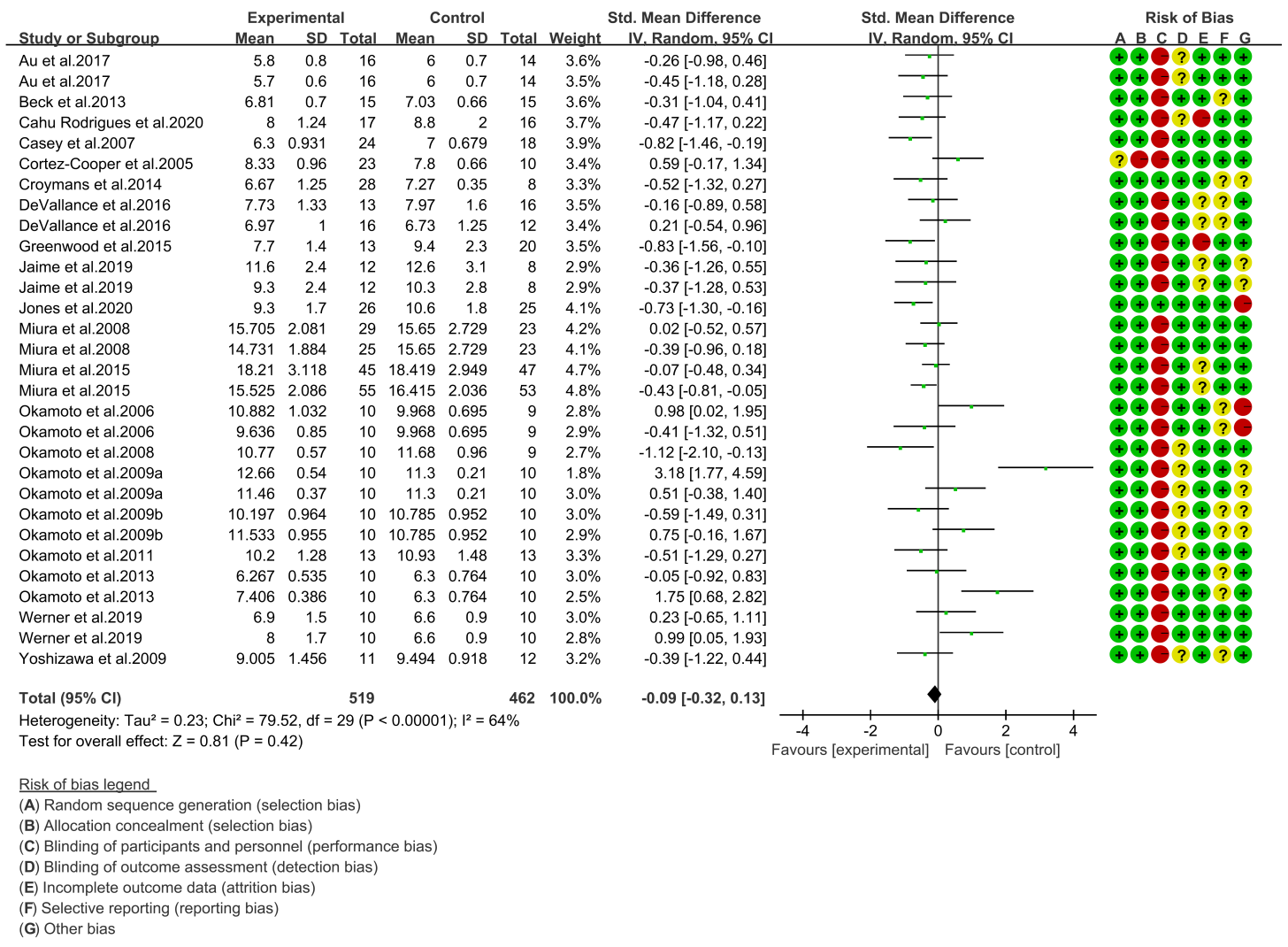

Supplement: Supplementary file 1 [file Data_Sheet_1.docx]
